# Building Tolerance Remotely: Telemedicine Support for Egg Ladder Completion in IgE‐Mediated Egg Allergy

**DOI:** 10.1111/cea.70303

**Published:** 2026-04-16

**Authors:** Mayah Cousens, Aoife Gallagher, Caoimhe Cronin, Sadhbh Hurley, Juan Trujillo

**Affiliations:** ^1^ Department of Paediatrics and Child Health University College Cork Cork Ireland; ^2^ Irish Centre for Maternal and Child Health Research (INFANT), HRB Clinical Research Facility Cork (CRF‐C) Cork Ireland; ^3^ Department of Paediatrics Cork University Hospital, Wilton Cork Ireland

## Abstract

- Hybrid telemedicine follow‐up achieved outcomes comparable to fully in‐person care without affecting duration or success.
- Telemedicine maintained treatment continuity during COVID‐19 disruption, preserving outcomes while reducing in‐person visits.

To the Editor,

Egg allergy is a common cause of food allergy and anaphylaxis in infancy [1]. Although historically managed through strict avoidance, dietary advancement therapy using structured food ladders allows gradual reintroduction of egg in progressively less heated forms, promoting sustained unresponsiveness [2]. In Ireland, this approach is implemented through the Irish Food Allergy Network (IFAN) Egg Ladder [3], with evidence supporting its safety, even in children with a history of anaphylaxis [4]. Since the COVID‐19 pandemic, telemedicine has facilitated remote guidance and follow‐up, improving accessibility and continuity of care. This study evaluated whether telemedicine‐supported follow‐up achieved outcomes comparable to traditional face‐to‐face care for egg ladder progression and completion in children with IgE‐mediated egg allergy.

This retrospective, single‐centre study at Cork University Hospital compared patients managed exclusively face‐to‐face (2015–2016) with those receiving telemedicine‐supported follow‐up (2021–2022). The earlier cohort was selected to ensure complete outcome classification before telemedicine introduction in 2020, and follow‐up for the telemedicine group extended through 2025 to allow adequate outcome assessment. Inclusion required a hen's egg skin prick test ≥ 3 mm or serum‐specific IgE > 0.35 kIU/L, in addition to a documented clinical history of reaction to egg. All participants underwent initial in‐person assessment with history, examination, and allergy testing (SPT and serum‐specific IgE). The telemedicine cohort had alternating telephone and in‐person follow‐up with additional in‐person review as required, while the in‐person cohort was managed entirely face‐to‐face.

Children older than 36 months at ladder initiation and those with incomplete data were excluded. Tolerance was defined as ingestion of > 30 mL raw egg (or equivalent) without symptoms; failure as inability to introduce raw egg within 36 months. Analyses were performed using SPSS v29. Given that telemedicine was implemented as a service adaptation to maintain care delivery, analyses were conducted within a non‐inferiority framework to assess whether telemedicine‐supported follow‐up achieved outcomes comparable to traditional face‐to‐face care. Informed consent was waived due to the retrospective use of anonymised clinical data.

Infants meeting inclusion criteria were identified from the paediatric allergy clinic between 2015 to 2016 and 2021 to 2022. A total of 77 patients were included in the final analysis (non‐telemedicine *n* = 44; telemedicine *n* = 33).

Infants receiving a hybrid of both telemedicine and face‐to‐face follow‐up showed no significant difference in ladder duration compared with fully in‐person follow‐up (20.32 vs. 18.20 months; *p* = 0.591). Completion rates were similarly high (84.8% hybrid vs. 81.8% face‐to‐face; *p* = 0.725), with no difference in treatment failure. These findings indicate that telemedicine‐supported care achieves outcomes comparable to traditional face‐to‐face management for egg ladder progression and completion, aligning with broader paediatric telemedicine evidence demonstrating similar outcomes, adherence, and patient satisfaction to in‐person care [5, 6]. Collectively, these results support telemedicine as a safe and effective adjunct for managing paediatric egg allergy, expanding access without compromising care quality.

Total appointment numbers were similar (*p* = 0.098), though visit intervals were shorter in the telemedicine group by 1.2 months (*p* = 0.001). In‐person patients averaged 2.8 visits at 6.8‐month intervals, whereas the hybrid group had 1.79 in‐person visits spaced 12.85 months apart (*p* = 0.001 for both), reducing reliance on face‐to‐face consultations while maintaining overall clinical contact. In combination with the findings that telemedicine had no impact on ladder duration or treatment completion, these findings suggest that the hybrid model can safely replace certain in‐person visits without compromising engagement or monitoring. Practically, this reduces travel, school/work disruptions, and improves access for children in rural or underserved areas [7], while maintaining adherence, supporting telemedicine as a sustainable component of paediatric allergy care [8].

Loss to follow‐up did not differ significantly between cohorts (10.8% telemedicine vs. 4.3% in‐person; *p* = 0.41). Notably, telemedicine was implemented during the COVID‐19 pandemic, when paediatric services in Ireland experienced reduced outpatient capacity and disrupted follow‐up [9]. Despite these pressures, attrition did not increase, suggesting hybrid care maintained continuity during service disruption.

Baseline characteristics were comparable between groups. The proportion of males (56.8% vs. 69.7%; *p* = 0.248) and mean age at diagnosis (15.30 months vs. 16.42 months; *p* = 0.556) did not differ significantly. Rates of atopic dermatitis (75.0% vs. 66.7%; *p* = 0.423), asthma/viral‐induced wheeze (15.9% vs. 6.1%; *p* = 0.183), cow's milk allergy (11.4% vs. 27.3%; *p* = 0.073), peanut allergy (20.5% vs. 18.2%; *p* = 0.803), and family history of atopy (70.5% vs. 51.5%; *p* = 0.09) were also similar (all *p* > 0.05). Skin prick test results and egg‐specific IgE levels (Figure 1A,B) were likewise comparable, indicating well‐matched cohorts at baseline. This reduces confounding and supports that similar ladder duration, completion, and follow‐up reflect comparable care models rather than baseline differences.

**FIGURE 1:**
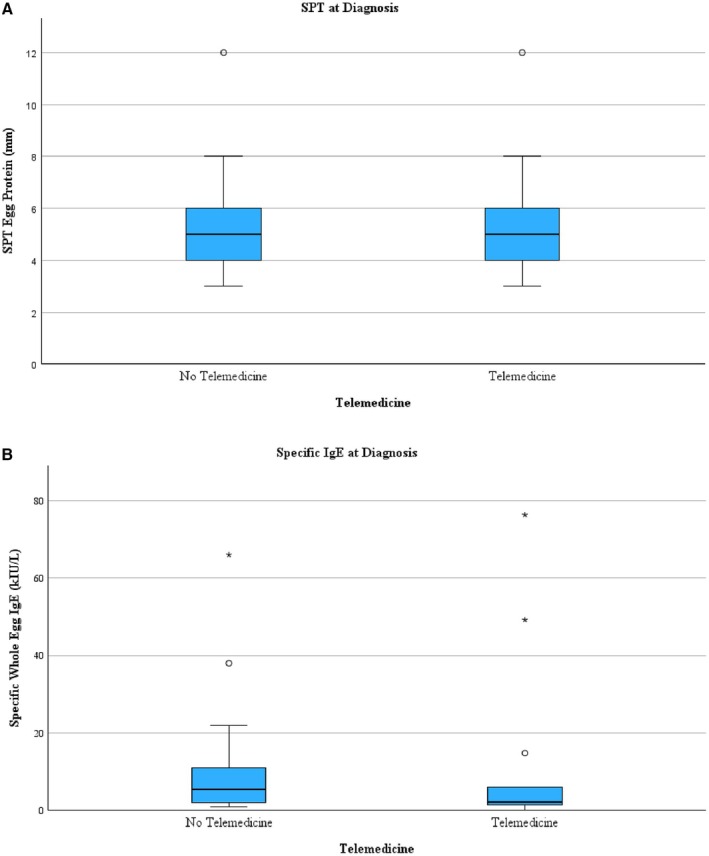
(A) Skin Prick Test (SPT) in the non‐telemedicine and telemedicine cohorts. (B) Specific IgE for egg at diagnosis between the two groups.

As a retrospective single‐centre study, findings are subject to documentation bias and potential residual confounding. Exclusion of patients lost to follow‐up may have favoured improved outcomes, though numbers were small. The modest sample size may also limit statistical power. However, the study was conducted in the primary tertiary paediatric allergy centre for the Munster region within a centralised national system using standardised egg ladder protocols, supporting cohort representativeness and generalisability within Ireland. Larger prospective studies are warranted.

In conclusion, hybrid telemedicine achieved outcomes comparable to fully in‐person care, without differences in treatment duration, completion, or loss to follow‐up. Introduced in response to reduced face‐to‐face access during the COVID‐19 pandemic, telemedicine has evolved into an effective model that maintains treatment delivery without increasing healthcare resource utilisation. These findings support the safe integration of telemedicine as an adjunct to in‐person care in paediatric egg allergy management.

## Author Contributions

**Mayah Cousens:** conceptualization, methodology, validation, data curation, formal analysis, writing – original draft preparation, visualisation. **Aoife Gallagher:** conceptualization, methodology, validation, resources, data curation, writing – review and editing, supervision. **Caoimhe Cronin:** resources, data curation, writing – review and editing. **Juan Trujillo:** conceptualization, methodology, validation, resources, writing – review and editing, supervision.

## Funding

The authors have nothing to report.

## Disclosure

Medical Writing/Editorial Assistance: No external or internal medical writing or editorial assistance was used in the preparation of this manuscript.

## Ethics Statement

Ethical approval for this retrospective study was obtained from the Cork University Hospital Research Ethics Committee (ECM 4 (y) 11/01/2022 and ECM 3 (yy) 10/09/2024).

## Conflicts of Interest

The authors declare no conflicts of interest.

## Data Availability

The data that support the findings of this study are available on request from the corresponding author. The data are not publicly available due to privacy or ethical restrictions.
